# Global Gene Expression Profiling in Whole-Blood Samples from Individuals Exposed to Metal Fumes

**DOI:** 10.1289/txg.7273

**Published:** 2004-11-22

**Authors:** Zhaoxi Wang, Donna Neuburg, Cheng Li, Li Su, Jee Young Kim, Jiu Chiuan Chen, David C. Christiani

**Affiliations:** ^1^Department of Environmental Health, Occupational Health Program, Harvard School of Public Health, Boston, Massachusetts, USA; ^2^Department of Statistical Sciences, Dana Farber Cancer Institute, Harvard Medical School, Boston, Massachusetts, USA; ^3^Pulmonary and Critical Care Unit, Department of Medicine, Massachusetts General Hospital, Harvard Medical School, Boston, Massachusetts, USA

**Keywords:** functional pathway, gene expression profiling, inflammation, occupational particulate exposure, whole-blood total RNA

## Abstract

Accumulating evidence demonstrates that particulate air pollutants can cause both pulmonary and airway inflammation. However, few data show that particulates can induce systemic inflammatory responses. We conducted an exploratory study using microarray techniques to analyze whole-blood total RNA in boilermakers before and after occupational exposure to metal fumes. A self-controlled study design was used to overcome the problems of larger between-individual variation interferences with observations of relatively smaller changes caused by environmental exposure. Moreover, we incorporated the dichotomous data of absolute gene expression status in the microarray analyses. Compared with nonexposed controls, we observed that genes with altered expression in response to particulate exposure were clustered in biologic processes related to inflammatory response, oxidative stress, intracellular signal transduction, cell cycle, and programmed cell death. In particular, the preinflammatory cytokine interleukin 8 and one of its receptors, chemokine receptor 4, seemed to play important roles in early-stage response to heavy metal exposure and were down-regulated. Furthermore, most observed expression variations were from nonsmoking exposed individuals, suggesting that smoking profoundly affects whole-blood expression profiles. Our study is the first to demonstrate that with a paired sampling study design of pre- and postexposed individuals, small changes in gene expression profiling can be measured in whole-blood total RNA from a population-based study. This technique can be applied to evaluate the host response to other forms of environmental exposures.

Exposure to ambient particulate air pollution is associated with increases in morbidity and mortality from respiratory and cardiovascular diseases (Godleski et al. 2000). The welding process generates high levels of metal fume containing respirable particles. Epidemiologic studies have shown that acute exposure to welding fume is associated with metal-fume fever (Mueller and Seger 1985) and increased reversible respiratory symptoms (El-Zein et al. 2003a; Wolf et al. 1997). There was an increased prevalence of inflammatory lung diseases, such as asthma and chronic bronchitis, among welders (El-Zein et al. 2003b). Additionally, accumulating epidemiologic evidence in the last decade has pointed to the associations of particulate exposure with adverse cardiovascular effects (Dockery et al. 1993; Mann et al. 2002; Peters et al. 2000, 2001a; Pope et al. 2002). Limited evidence indicates that welding-fume exposure also may be associated with increased cardiovascular events (Sjogren et al. 2002).

It has been proposed that inhaled particulates from air pollution may cause systemic alterations by the release of inflammatory cytokines subsequent to pulmonary inflammation, which plays an important role in the pathogenesis of atherosclerosis and coronary diseases. Indeed, elevated ambient particulate levels have been shown to be associated with increased levels of inflammatory markers, such as white blood cell (WBC) counts (Schwartz 2001), C-reactive protein (CRP; Peters et al. 2001b; Seaton et al. 1999), and fibrinogen (Pekkanen et al. 2000; Schwartz 2001) in both cross-sectional and longitudinal epidemiologic observations. In the experimental setting, animal studies have revealed that concentrated ambient particulate exposures increase the total WBC counts and the differential count of circulating neutrophils (Clarke et al. 2000; Gordon et al. 1998) in both healthy animals and those with pulmonary hypertension. Intratracheal instillation of residual oil fly ash (ROFA) can induce a significant elevation of plasma fibrinogen in cardiopulmonary-compromised rats (Gardner et al. 2000). Suwa et al. (2002) in their important work showing progressive atherosclerosis related to particulate exposure in hyperlipidemic rabbits also noted an increase in circulating polymorphonuclear leukocyte counts caused by exposures to particulate matter (PM) with a mass median aerodynamic diameter ≤10 μm (PM_10_).

However, most previous studies evaluated only downstream markers for systemic inflammatory responses. Direct human evidence is still lacking that shows particulates can induce systemic inflammation, although previous human studies and animal experiments did generate data, suggesting the involvement of inflammatory responses in particulate-mediated acute cardiac events. If particulate-mediated systemic inflammation were responsible for the observed adverse effects on the cardiovascular system, we would expect to see corresponding changes in mRNA expression for particulate-mediated systemic inflammation. The study described in this article addresses this mechanistic gap by investigating the systemic inflammatory response to welding-fume exposure using cDNA microarray technology on whole-blood total RNA. Blood samples were collected from welders and nonwelding controls before and after the work shift. We hypothesized that welding-fume exposure would be associated with systemic inflammation, as indicated by the findings that genes involved in systemic inflammation have significantly altered expressions. Furthermore, previous epidemiologic studies have shown that cigarette smoking significantly affects CRP, fibrinogen, and WBC levels (Frohlich et al. 2003; Smith et al. 2003). Therefore, we also hypothesized that smoking status would significantly affect the association between welding fume and the various systemic inflammatory gene expressions.

Microarray technology provides a format for the simultaneous measurement of the expression of thousands of genes in a single experimental assay and quickly becomes one of most the powerful and versatile tools for genomics and biomedical research (Murphy 2002). Peripheral blood is an essential tissue type for biomedical and clinical research because of its critical roles in immune response and metabolism. Furthermore, considering the simplicity and ease of collection, peripheral blood is also essential for discovery of biomarkers of hematologic diseases and surrogate markers of a wide range of nonhematologic disorders. Thus, applying microarray technology on peripheral blood may provide new insights of variations in global gene expression specifically associated with states of normal and disease and has the potential of applying the technology in disease detection and diagnosis. However, with the challenges unique to the blood sample, including complex composition of heterogeneous cell types and *ex vivo* changes of expression profiles induced by different handling and processing methods, it is difficult to apply microarray technology on whole-blood total RNA, and there are few previous publications of such research. To this end, this study is also an exploratory research with the purpose of developing proper methods for applying microarray technology on whole-blood total RNA.

## Materials and Methods

### Study population.

The study was approved by the institutional review board of the Harvard School of Public Health, and written informed consent was obtained from each subject. The study population consisted of 28 welding apprentices, instructors, and union officers, recruited and monitored at an apprentice welding school (Union Local 29, Quincy, MA). All 18 exposed subjects actively welded in the workshop, whereas 10 nonexposed controls stayed in the office or classroom of the same building during the work period. Blood samples were collected from each subject before and after the welding workshop. A self-administered questionnaire was used to obtain relevant information, including respiratory symptoms and diseases, smoking history, and occupational history. Exposure to fine particulate matter (particulate matter with a mass median aerodynamic diameter ≤2.5 μm, PM_2.5_) was assessed using KTL cyclones (GK2.05SH; BGI Inc., Waltham, MA). The air sample was collected on a 37-mm polytetrafluoroethylene membrane filter (Gelman Laboratories, Ann Arbor, MI), and the mass concentration was determined as previously described (Kim et al. 2003).

### Blood measurements.

Complete blood counts of all blood samples were carried out at Path Lab Inc. (Portsmouth, NH). The blood parameters included total WBC count with differential, red blood cell count, platelet count, hemoglobin, hematocrit, and erythrocyte indices (mean corpuscular volume, mean corpuscular hemoglobin, mean corpuscular hemoglobin concentration, and red cell distribution width).

### RNA preparation.

Immediately after the blood was drawn, we added TRI Reagent BD (Molecular Research Center, Inc., Cincinnati, OH) and mixed to stabilize the whole-blood total RNA. The stabilized samples were transported to our laboratory on dry ice and stored at −80°C until RNA extraction. Total RNA was isolated later from 10 mL of whole blood according to manufacturer protocols and purified using the RNeasy mini kit (Qiagen, Chatsworth, CA). The yield and quality of RNA were assessed by spectrophotometry and the Agilent 2100 Bioanalyzer (Agilent Technologies, Palo Alto, CA).

### Microarray hybridization.

For genomewide expression profiling, we used Affymetrix Human Genome U133A GeneChips (Affymetrix, Santa Clara, CA), which allow detection of approximately 22,215 gene expression probe sets. All RNA samples were sent to and analyzed at the Microarray Core Facility of the Dana-Farber Cancer Institute (Boston, MA), according to the manufacturer’s manual. The baseline and postexposure RNA samples from each subject were processed together in one batch of microarray analysis to minimize inherent variations. The quality of microarrays analysis was initially assessed by examination of the 3′ to 5′ ratios of five housekeeping controls on U133A GeneChips.

### Normalization and data extraction.

We used DNA-Chip Analyzer 1.3 (dChip; http://www.dchip.org/) software to normalize the raw microarray signals and then calculate the model-based expression values using a default perfect-match–only model with outlier detection. dChip software applied an invariant set normalization method, which chose a subset of perfect-matched probes with small within-subset rank difference in the two microarrays to serve as the basis for fitting a normalization curve (Li and Wong 2001a, 2001b). The outlier detection algorithm of the dChip software allowed further quality assessment of microarray data by cross-referencing one array with other arrays through a modeling approach to identify problematic arrays (Li and Wong 2001b). To have a better fit in the model for more precise estimations of expression values, we included 10 additional microarrays in data normalization and extraction. The detection of whether a gene was expressed (present) or not expressed (absent) in a RNA sample was carried out by Affymetrix Microarray Suite (MAS) 5.0 software (Affymetrix) using one-sided Wilcoxon’s signed-ranked algorithm (Detection Calls; Liu et al. 2002).

### Microarray data analysis.

Initially we evaluated gene expression changes by comparing the large fold-changes of expression values between baseline and postexposed microarrays in both exposed (welders) and nonexposed (controls) subjects. Then, we focused on using the paired *t*-test in the dChip software package to flush out genes with small expression changes in response to metal particulate exposure. The results of the paired *t*-test were adjusted by standard errors associated with each gene expression value. Because dChip software only gave an expression value to each gene on an array without discriminating whether the gene was expressed, we attempted to incorporate the Detection Calls information generated from the Affymetrix MAS 5.0 software package in the data analyses.

### Hierarchical clustering.

The analyses were carried out by dChip software using a hierarchical clustering algorithm (Eisen et al. 1998) with average-linkage method. After linear transformation to standardize the expression values across all selected samples, the distance between two genes was calculated as 1-absolute standard correlation coefficient and was used in the subsequent repeated process to build phylogenetic tree of genes and samples.

### Clustering analyses using gene ontology.

Testing for significant change in a single gene is difficult to accomplish given the stringent criteria for significance in the multiple-test background of > 20,000 probe sets. Therefore, we focused instead on assessing the biologic functions enriched with genes identified from the paired *t*-test, using the annotations defined by the Gene Ontology Consortium (GO; http://www.geneontology.org/; Hakak et al. 2001). The GO annotations are structured, controlled vocabulary for describing the roles of genes in any organism. The probability of observing a particular number of genes in one GO biologic process (bioprocess) was tested using hypergeometric distribution as previously described (Tavazoie et al. 1999). Briefly, we addressed the problem as what is the probability of observation of at least (*x*) genes of a certain GO bioprocess annotation in a list of (*k*) genes from paired *t*-test results, given the background that there are (*n*) genes with the same GO annotation from total (*m* ) genes (total annotated genes or a subclass of GO annotated genes on the entire Affymetrix array). The *p*-values were calculated using the following formula:


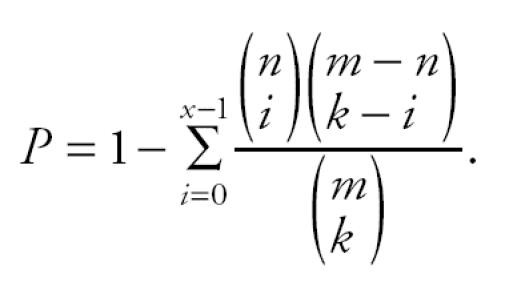


In this study, we focused only on the gene annotations of GO biologic process and used Affymetrix nonredundant build of human GO annotations downloaded 18 May 2004. The lists of genes were uploaded to Affymetrix NetAffx Analysis Center (http://www.affymetrix.com/analysis/index.affx) to obtain the numbers of genes within each GO bioprocess.

A potential problem of significance testing using GO annotations is that the hypergeometric distribution *p*-values are biased and sensitive to the total genes (*m*) used in the tests, which are not truly representing the entire genome because of the selection biases in array design and incomplete process of GO annotation. Furthermore, the problem of multiple testing is difficult to adjust because the GO bioprocesses are highly interrelated and genes are often assigned into multiple GO bioprocesses. Because GO has a multiple-level structure of directed acyclic graphs with each level of bioprocess linked through multiple parent–child relationships, there are one or more pathways that could be identified by tracing back from any GO bioprocess to the top using true-path–rule logic relationships. Thus, we adopted a conservative approach of testing the hypergeometric distributions. First, we used three numbers of the total genes (*m*) corresponding to the top three levels of GO bioprocess. A GO bioprocess was regarded as significant when it had a *p* < 0.005 at the lowest testing level and a *p* < 0.05 at the immediate upper level. A functional pathway was regarded as significant when it had three consecutive GO bioprocesses tested significantly or had two consecutive significant bioprocesses but also tested significantly in other pathway(s). The results were visualized using GoSurfer Soft Mining Tool (https://www.affymetrix.com/analysis/query/go_analysis.affx).

### Statistical analysis.

Statistical analyses were performed using SAS version 6.12 (SAS Institute Inc., Cary, NC). Exposure status was dichotomized as nonexposed controls and welders. Study population characteristics between controls and welders were compared using two-sample *t*-tests, Wilcoxon rank-sum tests with exact *p*-values, and Fisher’s exact test. The mean (SD) values of the PM_2.5_ concentrations were determined for controls and welders. Wilcoxon rank-sum tests with exact *p*-values were performed to compare the PM_2.5_ concentrations in controls and welders and also in smokers and nonsmokers. To account for the repeated measurements, linear mixed models with an interaction term for exposure status and smoking status were used for analysis. A generalized autoregressive covariance structure was used to account for the exponential decay of the correlation function as the interval between the measurements increases (Verbeke and Molenberghs 1997). Restricted maximum likelihood was used to estimate the covariance parameters. Baseline mean (SEM) levels of systematic inflammatory markers in peripheral blood were calculated in controls and welders according to smoking status. Linear mixed models were used to compare the baseline levels of the systemic inflammatory markers in controls and welders and in smokers and nonsmokers. The effect of age on the baseline levels of the systemic inflammatory markers also was investigated. The level of significance for all analyses was set at 0.05.

## Results

### Study population characteristics.

A combined approach of using intraindividual (self-pairing samples) and interindividual controls was implemented in the study to *a*) minimize the biologic variability among individuals and *b*) compare more precisely the gene expression profiles of different exposure states. Eighteen welders and 10 nonexposed controls at an apprentice welding school were recruited, and blood samples were collected at two time points: baseline and after 5.8 ± 0.6 hr of exposure to welding fume. After microarray hybridization, we had complete data sets on 44 arrays from 15 welders and 7 controls available for subsequent analysis. Among the 6 excluded study subjects, 1 withdrew during the study, 4 had low yield or poor quality of RNA extraction, and 1 had a poor quality of microarray hybridization. Population demographic data are summarized in Table 1. All study subjects were male, including 18 Caucasians, 3 Hispanics, and 1 African American. The age and smoking status were comparable between exposed and control groups.

During the welding workshop, the welders were exposed to metal fume and airborne PM from shielded metal arc welding, gas tungsten arc welding, plasma arc cutting, and grinding, with carbon steel being the most commonly used base metal. The controls were exposed primarily to ambient levels of PM while performing bookwork and office tasks at the welding school. In this study, particulate samples were collected from all controls and welders. With comparable mean sampling times between controls and welders, the median PM_2.5_ concentrations of welders were significantly higher than those of nonexposed controls (*p* < 0.01). However, there were no significant differences in PM_2.5_ concentrations according to smoking status in welders (*p* = 0.9). Previous occupational exposures, as measured by years of boilermaking, were not significantly different between controls and welders (Table 1). Moreover, before the day of sample collection, all controls and 12 of 15 welders had at least a 5-day period of non-welding or nonboilermaking to wash out the effects of previous metal-fume exposure. Among three welders with shorter than 5-day washout periods, two performed welding 1 day before the welding workshop and one performed welding 2 days before the workshop.

### Systemic inflammatory marker levels in peripheral blood.

All study subjects, including both controls and welders, had their blood cell counts within the normal ranges and had similar baseline profiles of major systematic inflammatory markers. Controls and welders were not found to have significantly different mean baseline CRP (*p* = 0.4), fibrinogen (*p* = 0.8), absolute neutrophil count (*p* = 0.1), and absolute WBC counts (*p* = 0.1). However, smokers were found to have significantly higher mean baseline WBC (*p* < 0.01) and neutrophil (*p* < 0.001) levels than nonsmokers, among welders as well as in the entire studied population.

The changes of the systemic inflammatory markers across two time points were not significant in controls except for a significant increase of fibrinogen [25 mg/dL; 95% confidence interval (95% CI), 4–45] in the post-exposure measurements. In contrast, there was a significant increase in total WBC counts (mean change, +1.2 ×10^3^/μL; 95% CI, 0.6–1.8) in nonsmoking welders but not in smoking welders (mean change, +0.3 × 10^3^/μL; 95% CI, −1.3 to 1.8). Relative and absolute neutrophil counts were also increased significantly in nonsmoking welders (*p* < 0.02) but not in smoking welders (*p* > 0.7). The change profiles of CRP levels were opposite those of WBC and neutrophil counts, with a significant increase in smokers (*p* = 0.02) and a nonsignificant change in nonsmoking welders (*p* = 0.4). Fibrinogen levels did not change significantly between postexposure and baseline in both smoking and nonsmoking welders (*p* ≥0.6). Overall, our observations of acute metal-fume exposure were consistent with previous epidemiologic findings that increased levels of inflammatory markers were associated with elevated ambient particulate levels (Pekkanen et al. 2000; Peters et al. 2001b; Schwartz 2001; Seaton et al. 1999)

### Finding genes with large expression variations by fold-change analysis.

Initially, we tried to find genes with large alterations of expressions between baseline and postexposed microarrays by comparing the large *n-*fold-changes of expression values in both exposed (welders) and nonexposed (controls) subjects. In both the welder and control groups, there was no gene with a 2-fold greater difference of the mean expression levels between baseline and postexposure arrays and an absolute difference > 50. Moreover, for each pair of baseline and postexposed arrays from the same subject, we found that few genes had large fold-changes (median number of genes, 20; range, 1–123) regardless of exposure status. In addition, the correlation coefficients of the raw expression values across entire probe sets were high between baseline and postexposed arrays from the same subject (median, 0.971; range, 0.949–0.988). These observations suggested that the real signals of changes in gene expression profiling in response to occupational metal exposure were very small, which could be the compound results of mixed cell types and large amounts of hemoglobin RNA in the whole-blood samples.

### *Identifying genes with altered expressions by paired* t*-test.*

When all 22,215 probes on the U133A GeneChip were included in the paired *t*-test, we found more genes (*p* < 0.05) in welders (533 genes from 546 probes) than in controls (86 genes from 88 probes) (Table 2). Considering the absolute gene expression status, we further found that probes identified by the paired *t*-test in controls had a larger proportion of noninformative probes (60.5%) that had absent calls assigned by the Detection Calls algorithm in every tested array compared with those in welders (47.3%). Regarding the entire set of probes on GeneChip, our data set had an overall 49.0% of noninformative probes among all baseline arrays. The initial observations suggested there were only random variations and no statistically significant changes in whole-blood expression between postexposed and baseline samples in individuals without metal particulate exposure. We then conducted a series of paired *t-*tests in several subsets of genes, which had Present calls in at least one, 10%, 25%, and 50% arrays. With the increase of Present calls, the number of genes identified by paired *t*-tests dropped, but the difference in the numbers of identified genes between welders and controls increased (Table 2). Taken together, consistent findings of more genes identified by paired *t*-tests in welders than in controls suggested there were alterations of global gene expression profiling in the whole-blood total RNA in response to acute metal-fume exposure. In addition, only one gene, RIO kinase 3 (*RIOK3*), was identified and down-regulated in both welders and controls.

### *Sample clustering using genes identified in paired* t*-tests.*

**Figure 1:**
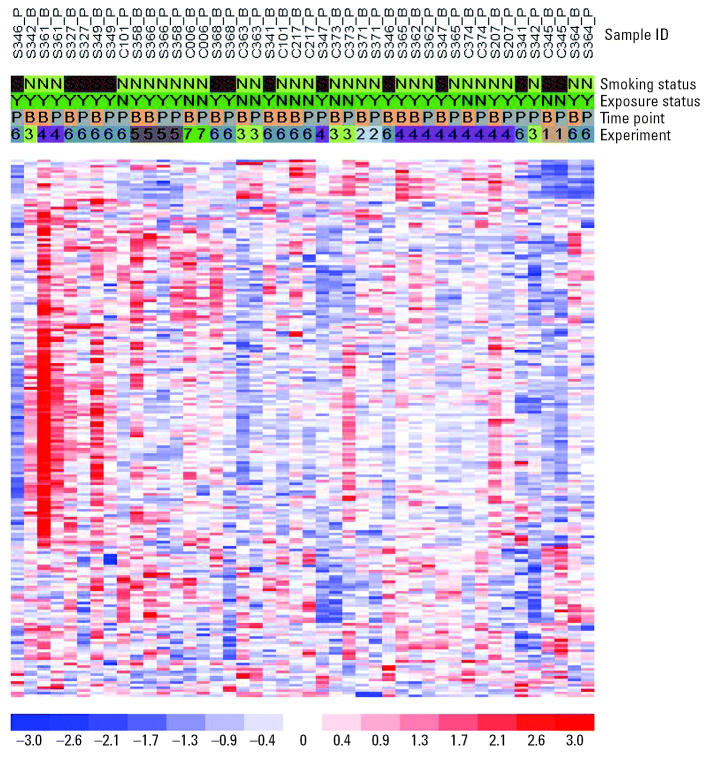
Cluster analysis 44 RNA samples using 139 genes identified by paired *t*-test in welders. The clustering display was generated by dChip software with two-way data clustering. Each row represents an individual gene, and each column corresponds to an individual array. Gene expression values were standardized and color coded relative to the mean: blue, values less than the mean; red, values greater than the mean. RNA samples from the same individual were labeled with the same sample ID with different suffixes, representing different collection time points. Smoking status: N, nonsmoking; S, smoking. Exposure status: N, controls; Y, welders. Time point: B, baseline; P, postexposure. Experiment: the number indicates the hybridization batch in which a sample was analyzed.

Genes identified by paired *t*-tests were used to classify RNA samples in hierarchical clustering analyses to further evaluate the expression patterns in samples categorized by different collection time points, smoking status, and metal-fume exposure status. We tested various lists of genes obtained from paired *t*-tests in controls, welders, nonsmoking welders, and smoking welders on the original expression data of baseline and postexposure arrays, as well as the data of log_2_-transformed expression ratios of postexposure over baseline. The clustering results neither revealed any distinct pattern of gene expressions with any kind of combination of selected genes and RNA samples nor showed any subgroup of samples or genes with similar expression patterns. However, in general, we found that > 70% of samples had the baseline and postexposure arrays of the same individual always clustered next to each other, regardless their exposure status (Figure 1). When RNA samples of non-smoking controls and welders were clustered with genes identified from paired *t*-tests of nonsmoking welders, all study participants had their baseline and postexposure arrays grouped together in the phylogenetic tree of sample clustering. Furthermore, samples of controls and welders seemed to be randomly mixed in any sample clustering analyses, including those using the data of log_2_-transformed expression ratios (data not shown). These observations further demonstrate that the real signals of gene expression changes caused by occupational metal exposure were smaller than the interindividual variations.

### Functional clustering using gene ontology.

**Figure 2:**
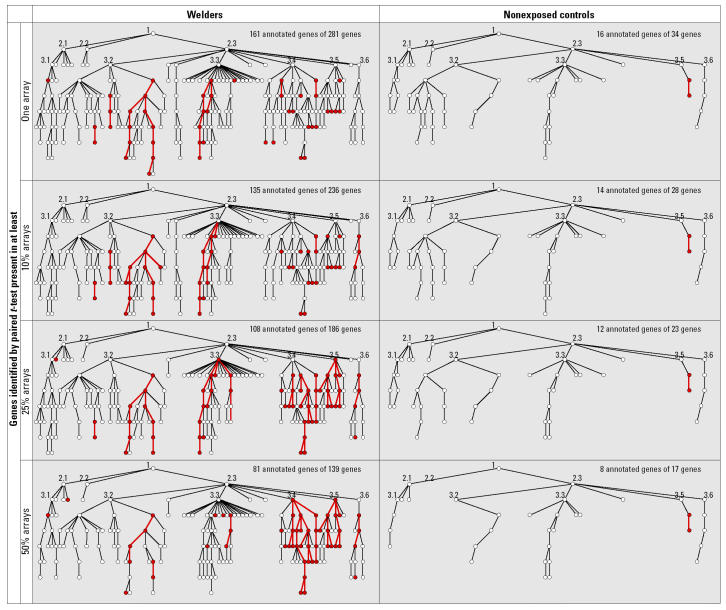
GoSurfer graphic view of hypergeometric distribution testing of gene clustering. Each node represents a GO biologic process, and a line connecting nodes represents parent–child relationship in the top-down direction. Because GO allows multiple parent–child relationships toward one biologic process but GoSurfer only plots one upstream and one downstream relationship for each node, one biologic process may appear several times in the GoSurfer plot. Red nodes represent significant GO bioprocesses tested by hypergeometric distribution as described in “Materials and Methods.” Numbered GO bioprocesses were used in calculation in hypergeometric distribution testing: 1, biologic process; 2.1, cellular process; 2.2, development; 2.3, physiologic processes; 3.1, cell communication; 3.2, cell growth and/or maintenance; 3.3, metabolism; 3.4, response to external stimulus; 3.5; response to stress; and 3.6, death.

Next, the genes identified by paired *t*-tests were evaluated by hypergeometric distribution testing based on GO annotations to define any bioprocesses enriched with the identified genes. To minimize the noise of the false-positive genes on the paired *t*-test, we applied a set of highly stringent criteria to define the significant GO bioprocesses and functional pathways and further observed the trends and distribution of the significant bioprocesses in four subsets of genes, with increasing percentage of Present calls among all arrays (at least one, 10%, 25%, and 50% arrays). The results are shown in Figure 2. With a decrease in the available numbers of genes and an increase in the percentages of Present calls, the main structures of GO bioprocesses were preserved in both welders and controls except for a few low-level bioprocesses that disappeared. In the nonexposed group, we did not find that genes were significantly enriched in any functional pathways except for two statistically significant bioprocesses: response to DNA damage stimulus (GO ID 6974) and nucleotide-excision repair (GO ID 6289). These two bioprocesses also existed in the welders but were not statistically significant. However, in contrast to the controls, in the welder group many GO bioprocesses were found to be significantly enriched with genes having significant alterations of expression after exposure to metal fume. Some of the GO bioprocesses tested significantly across all subsets with different Present calls. In subsets including genes with lower Present calls, the significant bioprocesses were distributed more discretely, with fewer functional pathways identified. With the increase of Present calls, more significant functional pathways showed up in welders by connecting discrete bioprocesses with newly appeared ones. In the subset of at least 50% Present calls, most significant bioprocesses were in the interconnected functional pathways. In the metal-exposed welders, functional pathways related to nucleic acid metabolism (including RNA metabolism and DNA metabolism), and cellular morphogenesis disappeared with the increase of Present calls.

We identified eight functional pathways with significant enrichment of genes having altered expressions in response to metal-fume exposure in the subset of genes having Present calls in > 50% arrays (Table 3). These functional pathways contained many GO bioprocesses related to proinflammatory and immune responses, oxidative stress, phosphate metabolism, cell proliferation, and programmed cell death. Moreover, we identified 35 genes from these significant pathways that had altered expression levels in welding fume–exposed individuals in comparison with their own baseline samples (Table 4). Among the identified genes, we found several genes involved in every aspect of the inflammatory response, including proinflammatory mediators, cytokine receptors, downstream signal transduction genes, and cytotoxic granulysin.

### Smoking effects on gene expression profiling.

We assessed further the effects of smoking on acute particulate exposure expression profiles. Of 15 welders and 7 nonexposed controls, there were 6 smoking welders and 1 smoking control. It appeared that most observed expression alterations were from nonsmokers exposed to welding fume because the number of genes identified from the paired *t*-test and the cluster of genes in GO bioprocesses were comparable between this subgroup of welders and the entire welding group (Table 5). In contrast to nonsmoking welders, fewer genes were identified from the paired *t*-test in welding smokers, and they had different patterns of gene clustering. A similar finding was observed in the analysis of the peripheral WBC count as described in the preceding section, and our results suggest that smoking may alter expression profiles in whole-blood total RNA and is a confounding factor in the study of particulate exposure-induced gene expression profiling changes.

## Discussion

In the present study, small expression alerations in several genes, caused by short-term occupational exposure to metal particulates, could be detected in whole-blood total RNA by paired *t*-tests. Based on GO annotations, the significant genes were clustered in functional pathways related to proinflammatory and immune responses, oxidative stress, phosphate metabolism, cell proliferation, and programmed cell death, suggesting systemic reactions in peripheral blood in response to environmental particulate exposure. Moreover, the observations were confounded by smoking because most variations were observed in non-smoking welders exposed to welding fume.

Accumulating evidence proved that microarray technology for the investigation of global gene expression profiling is a powerful tool for basic biologic research and laboratory investigations of patient materials, especially in the field of cancer research and toxicology. Although this technology had been successfully applied on fractionated blood samples (Klein et al. 2001; Locati et al. 2002) such as peripheral blood mononuclear cells (PBMCs), successful studies of gene expression profiles in whole-blood total RNA have been limited because of the difficult challenges of heterogeneous cell types and potential *ex vivo* changes from blood handling and processing. Compared with fractionated blood samples, whole-blood total RNA had lower detection sensitivities mainly caused by a large amount of hemoglobin RNA from reticulocytes, which contributes up to 70% of the total RNA isolated from whole blood (Affymetrix 2003a, 2003b). PBMCs have a more uniform cell population, containing lymphocytes and monocytes but excluding red blood cells and granulocytes (eosinophils, basophils, neutrophils), and are the most transcriptionally active cells in blood (DePrimo et al. 2003). However, the extra fractionation procedure for PBMCs requires a prolonged period before RNA stabilization, which results in significant *ex vivo* changes in gene expression profiling (Affymetrix 2003a; Pahl and Brune 2002). In this study, because all blood samples were collected within 1 day, it was beyond the capacity of our laboratory to fractionate all blood samples in a timely fashion. Thus, the whole-blood total RNA was extracted and applied in all subsequent microarray assays.

Compared with person-to-person variations of gene expressions, the exposure-induced gene expression changes were smaller. Regardless of exposure status, a pair of baseline and postexposed microarrays of the same subject often had a higher correlation coefficient of raw signals across entire probe sets than a pair of baseline microarrays randomly selected, and most pairs were clustered next to each other in sample clustering analyses. In addition, excess hemoglobin RNA and mixed cell types in the whole blood made it more difficult to observe the real changes in gene expression profiles. Under such circumstances, we were able to control better the biologic variability among individuals and obtain more sensitive and precise measurements on gene expression profiles by using self-paired controls. In our experiments, this test identified more genes in the exposed group (139 genes) than in nonexposed controls (17 genes), with Present calls in at least 50% arrays.

Affymetrix U133A GeneChip contains > 20,000 probes for measuring gene expressions in a single hybridization experiment. One major issue in data analysis is to determine whether changes in gene expression are experimentally significant, with the background of thousands of individual genes tested simultaneously. On a GeneChip, many genes are functionally interrelated or have unknown functions, and there are multiple probe sets detecting the same gene. In addition, the weak signals of exposure-induced changes made it very difficult, or even impossible, to conduct a valid multiple testing adjustment. With these considerations, we did not perform any adjustments on the results of the paired *t*-test in the present study.

An alternative approach in the statistics of multiple testing is to estimate the false discovery rate (FDR) by random permutations within the same data set (Tusher et al. 2001). We estimated the FDR of paired *t*-test results by permutating each pair of baseline and postexposed arrays 500 times using dChip 1.3 software. There was a lower FDR (median, 30.2%) in the exposed welders than in non-exposed controls (median, 112%), suggesting that the paired *t*-test results of the exposed group contained genes with real changes in expressions in response to occupational exposure. However, the permutation tests through dChip software did not adjust for the problem that multiple probe sets detect the same gene on a GeneChip, so the estimated FDRs could be inflated. Nevertheless, knowing that an approximately 30% FDR was associated with a set of genes from the paired *t*-test limits our ability to identify individual genes with statistically significant changes in expression in response to particulate exposure. Instead of further testing the significant change in a single gene, we focused on identifying significant pattern changes of biologic process in the genes identified from the paired *t*-test, using the annotations defined by GO. The underlying hypothesis is that several genes of one functional bioprocess change their expressions in response to environmental challenge because genes are highly networked and coordinated and do not act alone. Although one gene change may be small and difficult to be detected accurately in a significance test, the significant enrichment of genes with small changes in a biologic process and a functional pathway may be assessable.

In this study we identified 35 genes from eight significant functional pathways that had altered expression levels after metal-fume exposure. The most interesting finding was the identification of several genes involved in every aspect of the inflammatory response, including proinflammatory mediators, cytokine receptors, downstream signal transduction genes, and cytotoxic granulysin. Five genes (*IL8*, *IL1A*, *CXCR4*, *RALBP1*, and *SCYE1*) have been implicated in chemotaxis of the early inflammatory response, especially *IL8*, which is a critical mediator for neutrophil-dependent acute inflammation (Mukaida 2000, 2003). *IL8* has a wide range of actions on different cell types, including neutrophils, lymphocytes, monocytes, endothelial cells, and fibroblasts. *IL8* is produced from various cell types in response to a wide variety of stimuli, including proinflammatory cytokines, microbes and their products, and environmental changes such as hypoxia, reperfusion, and hyperoxia. Previous studies on ROFA-exposed workers found an increase in proinflammatory cytokines and polymorphonuclear cells in the nasal lavage fluid, indicating that the particulate exposure resulted in acute upper airway inflammation (Hauser et al. 1995; Woodin et al. 1998). In our study, *IL8* and other cytokines and receptor genes were transcriptionally down-regulated in whole-blood total RNA in response to metal particulate exposure.

Our findings that genes with altered expressions in whole-blood total RNA in response to metal particulate exposure were clustered in the functional pathways related to inflammatory and immune responses support the hypothesis that particulates induce systemic inflammation. It has been well documented that particulate air pollutants can cause both pulmonary parenchymal (Nel et al. 2001; Pope 2000) and airway inflammation (Peden 2001). These particulate-mediated local inflammatory responses conform to those epidemiologic observations that exposure to particulate air pollutants can lead to asthma exacerbation, increased pulmonary infections, decreased pulmonary functions, increased hospitalizations due to pulmonary and/or airway diseases, and increased mortality. Recent studies using high-throughput technology for gene expression profiling have added to our understanding of particulate-mediated local inflammation underlying those adverse effects on lungs and airway in response to air pollution. Increased RNA expression for stress response, inflammatory, and repair-related genes were observed in Sprague-Dawley rats after intratracheal instillation of ROFA (Nadadur and Kodavanti 2002). In co-cultures of alveolar macrophages and primary human bronchial epithelial cells, mRNA levels of tumor necrosis factor (*TNF*)-α, granulocyte macrophage colony stimulating factor (*GM-CSF*), interleukin *IL1*β, *IL6*, and *IL8* were increased within 2 hr (*p* < 0.05) after exposure to 100 μg/mL of PM_10_ (Fujii et al. 2002), and mRNA levels of leukemia inhibitory factor (*LIF*), *GM-CSF*, *IL1*α, and *IL8* in primary human bronchial epithelial cells were increased after exposure to PM_10_ (Fujii et al. 2001).

In this study, we also demonstrated that it was critical to apply a dichotomous definition of absolute gene expression status, that is, expressed versus nonexpressed, in the data mining of the microarray data. Many algorithms currently used for microarray analysis retrieve the expression data from raw signals as continuous data and do not distinguish the distinct dichotomous biologic status of a gene. If one gene was not expressed in a RNA sample, there was always a meaningless expression value being generated that could not be distinguished accurately from other samples that expressed the same gene. In reality there should be no mRNA in a sample when a gene is not expressed. If the expression values generated by an algorithm truly represented reality, the data for expressed and nonexpressed genes should have different distributions. Therefore, without distinguishing expression status, a large number of meaningless data from nonexpressed genes would have deteriorating effects on a statistical analysis that assumed a normal distribution of data. Our observations that more functional pathways were associated with high content of Present calls in welders support this hypothesis. Furthermore, based on the absolute expression status, microarray data may be divided into three categories: consistently not expressed, turned on or off, and continuously expressed in different experimental conditions. The first category of genes was noninformative, and the analyses of the second category of genes were very complicated and difficult. Only the last category of genes, those with a high percentage of Present calls across all arrays, was suitable for parametric statistical analysis. At present, the Detection call algorithm of Affymetrix MAS 5.0 is the only one available for determining the absolute gene expression status, with limitations on both sensitivity and specificity to distinguish low-level expressed genes from nonexpressed genes (Liu et al. 2002).

In conclusion, using a repeated measure design, peripheral blood gene expression profiles revealed that environmental exposures to metal fume in healthy individuals produced observable changes in gene expression clustered in biologic processes related to inflammatory, oxidative stress, phosphate metabolism, cell proliferation, and programmed cell death. Smoking modified the observed responses. Finally, our study demonstrates the utility of paired sampling pre- and postexposure in an at-risk population.

## Figures and Tables

**Table 1:** Demographics of study population.

	Welders	Nonexposure controls	*p-*Value
No. of subjects	15	7	
No. of smokers (%)	6 (40)	1 (14)	0.35^*^
Age, years	32 (22–46)	40 (19–57)	0.69^**^
Years of boilermaking	3 (2–20)	3 (1.5–33)	0.61^**^
Number with hypertension (%)	1 (7)	2 (29)	0.23^*^
Welding fume exposure (PM_2.5_ concentration, mg/m^3^)	2.44 (1.30–3.42)	0.04 (0.02–0.17)	< 0.001^**^

Unless specified, values are expressed as median (range) and were tested by the median test.

*Fisher’s exact test.

**Wilcoxon rank-sum test with exact *p*-value.

**Table 2:** Genes identified by paired *t*-test: postexposure versus baseline microarrays.

Gene with Present calls in	Welders (28 arrays/14 pairs)	Nonexposed controls (16 arrays/8 pairs)	Gene ratio (welders:controls)
All arrays	533	86	6.20
At least one array	281	34	8.26
At least 10% arrays	236	28	8.43
At least 25% arrays	186	23	8.09
At least 50% arrays	139	17	8.18

**Table 3:** Results of hypergeometric testing using annotations from the Gene Ontology Consortium (GO).*^a^*

				Genes from paired *t*-test*^b^*					
Functional pathway	GO ID	Biologic processes	Genes annotated on array	Welders	Controls
1	9605	Response to external stimulus	959	18	0
	42330	Taxis	88	5	0
	6935	Chemotaxis	88	5	0
	30595	Immune cell chemotaxis	2	1	0
	30593	Neutrophil chemotaxis	1	1	0
2	9605	Response to external stimulus	959	18	0
	9607	Response to biotic stimulus	653	15	0
	6952	Defense response	595	12	0
	6955	Immune response	548	12	0
	45087	Innate immune response	147	5	0
	6954	Inflammatory response	145	5	0
	42119	Neutrophil activation	1	1	0
	30593	Neutrophil chemotaxis	1	1	0
3	6950	Response to stress	616	16	2
	9605	Response to external stimulus	959	18	0
	9611	Response to wounding	213	7	0
	6954	Inflammatory response	145	5	0
	42119	Neutrophil activation	1	1	0
	30593	Neutrophil chemotaxis	1	1	0
4	9607	Response to biotic stimulus	653	15	0
	6950	Response to stress	616	16	2
	9613	Response to pest/pathogen/parasite	364	11	0
	6954	Inflammatory response	145	5	0
	42119	Neutrophil activation	1	1	0
	30593	Neutrophil chemotaxis	1	1	0
	9615	Response to viruses	28	2	0
5	9605	Response to external stimulus	959	18	0
	9607	Response to biotic stimulus	653	15	0
	6979	Response to oxidative stress	34	3	0
	6950	Response to stress	616	16	2
6	8219	Cell death	315	7	1
	12501	Programmed cell death	292	7	1
	6915	Apoptosis	291	7	1
	6916	Anti-apoptosis	60	3	1
7	8283	Cell proliferation	762	14	1
	7049	Cell cycle	491	14	1
	67	DNA replication and chromosome cycle	127	6	1
	84	S phase of mitotic cell cycle	100	4	0
	6260	DNA replication	99	4	0
	6270	DNA replication initiation	14	2	0
8	6793	Phosphorus metabolism	468	9	1
	6796	Phosphate metabolism	468	9	1
	16311	Dephosphorylation	78	4	0

a Genes were identified from paired *t*-test with Present calls in at least 50% arrays. Annotations are from Gene Ontology Consortium (http://www.geneontology.org/).

b All listed biologic processes tested significantly in hypergeometric distribution testing in welders but nonsignificantly in nonexposed controls.

**Table 4:** Genes with altered expressions in response welding-fume exposure.

			Welders		Controls	
Accession number*^a^*	Gene name*^a^*	Gene symbol*^a^*	Fold change	Paired *p*-value	Fold change	Paired *p*-value
NM_00584	Interleukin 8	*IL8*	−1.22	0.004	1.02	0.923
NM_000575	Interleukin 1, alpha	*IL1A*	−1.12	0.035	−1.04	0.673
NM_003467	Chemokine (C-X-C motif) receptor 4	*CXCR4*	−1.26	0.038	1.02	0.900
NM_004757	Small inducible cytokine subfamily E, member 1 (endothelial monocyte-activating)	*SCYE1*	−1.12	0.017	1.03	0.769
NM_006788	ralA binding protein 1	*RALBP1*	−1.16	0.036	−1.09	0.629
NM_004111	Flap structure-specific endonuclease 1	*FEN1*	−1.13	0.038	1.02	0.864
NM_000416	Interferon gamma receptor 1	*IFNGR1*	−1.39	0.014	−1.10	0.483
NM_078481	CD97 antigen	*CD97*	1.21	0.049	−1.00	0.930
NM_002339	Lymphocyte-specific protein 1	*LSP1*	1.21	0.040	1.14	0.225
NM_012483	Granulysin	*GNLY*	−1.18	0.034	1.05	0.577
NM_001766	CD1D antigen, d polypeptide	*CD1D*	−1.22	0.002	−1.09	0.394
NM_001828	Charot-Leyden crystal protein	*CLC*	−1.21	0.033	−1.18	0.540
NM_000633	B-cell CLL/lymphoma 2	*BCL2*	−1.12	0.010	1.08	0.198
NM_006144	Granzyme A (granzyme 1, cytotoxic T-lymphocyte-associated serine esterase 3)	*GZMA*	−1.27	0.045	1.00	0.985
NM_080549	Protein tyrosine phosphatase, non-receptor type 6	*PTPN6*	1.10	0.032	−1.05	0.612
NM_000345	Synuclein, alpha (non A4 component of amyloid precursor)	*SNCA*	−1.28	0.030	1.02	0.907
NM_002656	Pleiomorphic adenoma gene-like 1	*PLAGL1*	−1.20	0.039	−1.11	0.318
NM_201397	Glutathione peroxidase 1	*GPX1*	1.14	0.035	−1.01	0.897
NM_004417	Dual specificity phosphatase 1	*DUSP1*	−1.21	0.035	−1.09	0.331
NM_001752	Catalase	*CAT*	−1.22	0.044	−1.07	0.521
NM_004383	c-src tyrosine kinase	*CSK*	1.18	0.013	−1.02	0.736
NM_006999	Polymerase (DNA directed) sigma	*POLS*	−1.08	0.046	1.03	0.689
NM_002835	Protein tyrosine phosphatase, non-receptor type 12	*PTPN12*	−1.23	0.042	−1.02	0.810
NM_145906	RIO kinase 3 (yeast)	*RIOK3*	−1.25	0.008	−1.05	0.737
NM_181742	Origin recognition complex, subunit 4-like (yeast)	*ORC4L*	−1.13	0.032	−1.06	0.596
NM_052811	ret finger protein 2	*RFP2*	−1.13	0.037	1.03	0.750
NM_002577	p21 (CDKN1A)-activated kinase 2	*PAK2*	−1.20	0.020	1.03	0.751
NM_002848	Protein tyrosine phosphatase, receptor type, O	*PTPRO*	−1.14	0.041	−1.12	0.209
NM_015374	unc-84 homolog B (C. elegans)	*UNC84B*	1.12	0.044	−1.03	0.462
NM_004359	Cell division cycle 34	*CDC34*	1.17	0.013	−1.06	0.426
NM-002958	RYK receptor-like tyrosine kinase	*RYK*	−1.19	0.033	1.01	0.976
NM_014826	CDC42 binding protein kinase alpha (DMPK-like)	*CDC42BPA*	−1.21	0.020	1.04	0.843
NM_016839	RNA binding motif, single stranded interacting protein 1	*RBMS1*	−1.18	0.023	−1.09	0.535
NM_016113	Transient receptor potential cation channel, subfamily V, member 2	*TRPV2*	1.08	0.041	−1.07	0.208
NM_032454	Serine/threonine kinase 19	*STK19*	−1.11	0.044	1.07	0.456

a From Affymetrix NetAffx Analysis Center (http://www.affymetrix.com/analysis/index.affx).

**Table 5:** Effects of smoking on acute metal exposure expression profiles.

		Welders		Nonexposed controls	
	All welders	Nonsmokers	Smokers	All controls	Nonsmokers
Number of arrays	30	18	12	14	12
Paired *t*-test					
No. of genes with Present calls in all arrays	533	419	251	86	104
No. of genes with Present calls in at least 50% arrays	139	154	85	17	16
Hypergeometric distribution test in gene with Present call in at least 50% arrays*^a^*					
1*^b^* 9605*^c^* Response to external stimulus*^d^* (959)*^e^*	*18**^f^*	18*^f^*	8*^f^*	0*^f^*	0*^f^*
42330 Taxis (88)	*5*	3	1	0	0
6935 Chemotaxis (88)	*5*	3	1	0	0
30595 Immune cell chemotaxis (2)	*1*	*1*	0	0	0
30593 Neutrophil chemotaxis (1)	*1*	*1*	0	0	0
2 9605 Response to external stimulus (959)	*18*	18	8	0	0
9607 Response to biotic stimulus (653)	*15*	*16*	6	0	0
6952 Defense response (595)	*12*	*13*	6	0	0
6955 Immune response (548)	*12*	*13*	5	0	0
45087 Innate immune response (147)	*5*	3	0	0	0
6954 Inflammatory response (145)	*5*	3	0	0	0
42119 Neutrophil activation (1)	*1*	*1*	0	0	0
30593 Neutrophil chemotaxis (1)	*1*	*1*	0	0	0
3 6950 Response to stress (616)	*16*	*17*	4	2	2
9605 Response to external stimulus (959)	*18*	18	8	0	0
9611 Response to wounding (213)	*7*	4	1	0	0
6954 Inflammatory response (145)	*5*	3	0	0	0
42119 Neutrophil activation (1)	*1*	*1*	0	0	0
30593 Neutrophil chemotaxis (1)	*1*	*1*	0	0	0
4 9607 Response to biotic stimulus (653)	*15*	*16*	6	0	0
6950 Response to stress (616)	*16*	*17*	4	2	2
9613 Response to pest/pathogen/parasite (364)	*11*	*10*	3	0	0
6954op Inflammatory response (145)	*5*	3	0	0	0
42119 Neutrophil activation (1)	*1*	*1*	0	0	0
30593 Neutrophil chemotaxis (1)	*1*	*1*	0	0	0
9615 Response to viruses (28)	*2*	1	0	0	0
5 9605 Response to external stimulus (959)	*18*	18	8	0	0
9607 Response to biotic stimulus (653)	*15*	*16*	6	0	0
6979 Response to oxidative stress (34)	*3*	2	0	0	0
6950 Response to stress (616)	*16*	*17*	4	2	2
6 8219 Cell death (315)	*7*	*8*	5	1	1
12501 Programmed cell death (292)	*7*	*8*	5	1	1
6915 Aptosis (291)	*7*	*8*	5	1	1
6916 Anti-apoptosis (60)	*3*	*5*	2	0	0
7 8283 Cell proliferation (762)	*14*	*17*	5	1	1
7049 Cell cycle (491)	*14*	*14*	4	1	1
67 DNA replication and chromosome cycle (127)	*6*	*5*	1	1	1
84 S phase of mitotic cell cycle (100)	*4*	*5*	0	0	0
6260 DNA replication (99)	*4*	*5*	0	0	0
6270 DNA replication initiation (14)	*2*	1	0	0	0
8 6793 Phosphorus metabolism (468)	*9*	5	3	1	0
6796 Phosphate metabolism (468)	*9*	5	3	1	0
16311 Dephosphorylation (78)	*4*	2	0	0	0

a Italic numbers indicate that the functional pathway was tested significantly in hypergeometric distribution testing.

b Functional pathway.

c GO identification number (from GO Consortium: http://www.geneontology.org/)

d Biologic process.

e The number of genes on U133A array belonging to the functional pathway.

f The number of genes identified by paired *t*-test belonging to the functional pathway.
